# Influence and reliability of lower-limb arterial occlusion pressure at different body positions

**DOI:** 10.7717/peerj.4697

**Published:** 2018-05-02

**Authors:** Luke Hughes, Owen Jeffries, Mark Waldron, Ben Rosenblatt, Conor Gissane, Bruce Paton, Stephen D. Patterson

**Affiliations:** 1 School of Sport Health & Applied Science, St Mary’s University, Twickenham, UK; 2 St. George’s Park, The Football Association, Burton-Upon-Trent, UK; 3 Institute of Sport, Exercise and Health, University College London, University of London, London, UK

**Keywords:** Blood flow restriction exercise, Ischemic preconditioning, Occlusion, Limb occlusion pressure, Kaatsu

## Abstract

**Background:**

Total arterial occlusive pressure (AOP) is used to prescribe pressures for surgery, blood flow restriction exercise (BFRE) and ischemic preconditioning (IPC). AOP is often measured in a supine position; however, the influence of body position on AOP measurement is unknown and may influence level of occlusion in different positions during BFR and IPC. The aim of this study was therefore to investigate the influence of body position on AOP.

**Methods:**

Fifty healthy individuals (age = 29 ± 6 y) underwent AOP measurements on the dominant lower-limb in supine, seated and standing positions in a randomised order. AOP was measured automatically using the Delfi Personalised Tourniquet System device, with each measurement separated by 5 min of rest.

**Results:**

Arterial occlusive pressure was significantly lower in the supine position compared to the seated position (187.00 ± 32.5 vs 204.00 ± 28.5 mmHg, *p* < 0.001) and standing position (187.00 ± 32.5 vs 241.50 ± 49.3 mmHg, *p* < 0.001). AOP was significantly higher in the standing position compared to the seated position (241.50 ± 49.3 vs 204.00 ± 28.5 mmHg, *p* < 0.001).

**Discussion:**

Arterial occlusive pressure measurement is body position dependent, thus for accurate prescription of occlusion pressure during surgery, BFR and IPC, AOP should be measured in the position intended for subsequent application of occlusion.

## Introduction

The technique of occluding limb blood flow using pneumatic tourniquet cuffs is applied in various settings, such as during surgery (Bussani & McEwen, 1988), blood flow restriction exercise (BFRE) (Hughes et al., 2017) and ischemic preconditioning (IPC) (Griffin et al., 2017). The level of occlusion achieved by an applied pressure is considered to be an important factor for effective creation of a bloodless surgical field (Bussani & McEwen, 1988), driving physiological adaptations and preventing full occlusion of arterial blood flow during BFRE (Fahs et al., 2012; Lixandrão et al., 2015), and effectiveness of the IPC stimulus (Cunniffe et al., 2016). The required pressure to reach a desired level of occlusion is influenced by several factors, such as cuff width, limb circumference and blood pressure (Loenneke et al., 2015; Jessee et al., 2016), which makes standardisation of occlusion level difficult using arbitrary pressures. The use of arbitrary pressures in BFRE may influence the amount of fatigue observed during exercise and thus potential adaptations. Furthermore during IPC a standard pressure of 200–220 mmHg is widely used prior to exercise, irrespective of upper- or lower-limb application, which may influence the potential benefit of this technique due to different limb circumference and blood pressure within individuals (Bailey et al., 2012; Barbosa et al., 2014; Patterson et al., 2015). Calculation of arterial occlusive pressure (AOP) involves determination of the pressure required to fully occlude arterial flow to the involved limb (AORN Recommended Practices Committee, 2007). This is most often achieved using Doppler ultrasound (Bezerra de Morais et al., 2016) and can be used to prescribe pressure at a relative percentage of AOP to standardise the level of occlusion across cohorts (Laurentino et al., 2012; Hughes et al., 2017; Patterson et al., 2017).

Occlusion of blood flow is typically applied in one of three positions: supine, seated/semi-recumbent or standing (Loenneke et al., 2012). However, within the literature it is evident that only a small number of studies measure AOP in the same position that the occlusion stimulus is subsequently applied (Staunton et al., 2015); this may not account for postural influences on hydrostatic pressure (Wilkins, Halperin & Litter, 1950; Eiken, 1988). For example, movement of the lower-limb into a dependent position causes changes in hydrostatic pressure, deformation of the vascular bed and an increase in blood flow and pressure within the limb (Trinity et al., 2010). Systolic blood pressure has been identified as a major predictive variable of AOP in the upper limbs (Loenneke et al., 2015; Jessee et al., 2016), whereas thigh circumference is the largest predictor in the lower body (Loenneke et al., 2015), thus it is conceivable that posture-induced changes in blood flow and pressure may affect the pressure required for absolute occlusion of blood flow in that limb.

To date, only one study has investigated the influence of body position on AOP measurement (Sieljacks et al., 2018), however this was only in the supine and seated position. Furthermore the reliability of AOP in each of these body positions is unknown. This is problematic as it may result in under/over-estimation of the required pressure, which may have implications for effectiveness in application of an occlusion stimulus. Additionally, heterogeneous changes in AOP between individuals may lead to variation in occlusion stimulus even when prescribed relative to AOP. Thus, the aim of this study was to investigate the influence of different postural positions on AOP measurement. A further aim was to examine the test-retest reliability of AOP at different body positions.

## Materials and Methods

### Participants

Fifty participants (37 males and 13 females) volunteered to participate in the study. Overall mean ± SD for age, mass and stature were 29 ± 6 y, 77.3 ± 14.2 kg and 175.9 ± 8.4 cm, respectively. All were healthy, active non-smokers free from cardiovascular (CV), pulmonary and metabolic diseases and musculoskeletal injuries in the past 12 months. Participants were asked to refrain from strenuous exercise, caffeine and alcohol in the 24 h prior to each testing session. All participants provided signed informed consent in compliance with the Declaration of Helsinki, seventh version, October 2013 (World Medical Association, 2013). All protocols were approved by St Mary’s University ethical committee (SMEC_2016-17_121).

### Experimental design and procedure

To examine the influence of body position on AOP, participants attended the laboratory on one occasion. Upon arrival, participant’s mass and stature were recorded to the nearest 0.1 kg and 0.1 cm, respectively. Participants rested for 5 min in the supine position on a portable treatment bed, then blood pressure was measured at the brachial artery (Omron M5; Omron Healthcare, Europe B.V., the Netherlands, 14 × 48 cm). Thigh circumference (cm) of the dominant lower-limb was measured at the midpoint of the distance between the greater trochanter and the lateral condyle of the femur in accordance with International Society for the Advancement of Kinanthropometry guidelines using a flexible steel tape (Lufkin W606PM; Apex Tool Group, Sparks, MD, USA). Additionally, skinfold thickness (ST) was measured (mm) at this point using skinfold callipers (Harpenden skinfold callipers; British Indicators Ltd, Surrey, UK). For the experimental procedure, participants underwent AOP measurements in the dominant leg in a supine, seated and standing position in a within-subjects randomised design. The randomisation was carried out by assigning each participant a number and using publicly available software to allocate the order of conditions (http://www.randomization.com/). Prior to each measurement, participants rested in the required position for 5 min to ensure restoration of homeostasis after any movement. For the supine position, participants lay on a portable treatment bed with their arms relaxed by their sides. For the seated position, participants sat upright with their legs straight and the hip flexed at a 90° angle, assessed using a goniometer. For the standing position, participants stood in the standard anatomical position. Prior to each measurement, participants rested in the required position for 5 min to ensure restoration of normal blood flow after any movement (Jessee et al., 2016).

### Arterial occlusive pressure measurement

Restriction of blood flow in the lower-limb was achieved using the Delfi Easy-fit variable contour tourniquet cuff (11.5 cm × 86 cm × 5 mm), connected to a pneumatic cuff inflator (Delfi PTS, Delfi Medical, Vancouver, BC, Canada). The pneumatic tourniquet was equipped with the capability of automatically measuring AOP and calculating the personalised tourniquet pressure, comprised of a dual-purpose personalised tourniquet cuff and a personalised tourniquet instrument containing AOP calculation sensors and software. The pneumatic system connected to the tourniquet cuff increased the cuff pressure in stepwise increments, analysing the pneumatic pressure pulsations in the cuff bladder by the arterial pressure pulsations at each cuff pressure increment, and used these characteristics to determine AOP (McEwen et al., 2015). AOP measurement using this cuff was found to not be clinically or statistically different from using the gold standard Doppler technique (+0.08 mmHg (95% CI [−2.66 to 2.82]) for lower limbs) across 257 pairs of AOP measurements taken from upper and lower limbs in 143 pre- and post-surgical patients aged 17–86 (McEwen et al., 2015; Masri et al., 2016). This technique of measuring AOP was found to have clinically acceptable accuracy compared to the distal-sensor-based method of automatic AOP measurement, which measures AOP using a sensor located on the most distal phalange of the involved limb (McEwen, Inkpen & Younger, 2002). The variable contour cuff was placed on the most proximal portion of the participant’s dominant lower-limb directly onto the skin and connected to the pneumatic tourniquet with airtight hose tubing. After 5 min of rest, the device was turned on to calculate AOP in the manner described above. The AOP displayed on the pneumatic tourniquet device was recorded for each of the three positions.

### Test-retest reliability

To assess the reliability of the pneumatic tourniquet, 10 subjects visited the laboratory at the same time of day, on a second occasion, one week later during which the experimental procedure for the AOP measurement was repeated with the order of positions tested in the same order as they had previously been tested.

### Statistical analysis

Due to non-normal distribution of the supine body position data (*p* < 0.05) which persisted after log transformations, a non-parametric Friedman test was used to determine if there were differences in AOP across the three different body positions. For any significant differences, Wilcoxon signed-rank pairwise comparisons were performed with Bonferroni correction. Within-subject coefficient of variation (COV) was calculated, and an intraclass correlation coefficient (ICC) test with a two-way mixed effects model was used to determine absolute agreement to examine the reliability and reproducibility of AOP measurements with the pneumatic tourniquet system. The COV was calculated from the ratio of the standard deviation (SD) and the mean of the two AOP measurements (CV = ((SD/mean) × 100)), followed by calculation of the mean (Bezerra de Morais et al., 2016).

## Results

### Participants

All 50 participants completed the study with no adverse events. Participants’ blood pressure, resting heart rate, thigh circumference and ST are presented in Table 1.

**Table 1:** Participant anthropometric characteristics (Mean ± SD).

	Males (*n* = 37)	Females (*n* = 13)
Age (y)	29 ± 6	29 ± 7
Stature (cm)	179.9 ± 4.8	174.3 ± 7.9
Body mass (kg)	81.8 ± 13.3	72.7 ± 11.3
Systolic blood pressure (SBP; mmHg)	126 ± 9	123 ± 8
Diastolic blood pressure (DBP; mmHg)	72 ± 8	66 ± 17
Resting heart rate (bpm)	62 ± 9	61 ± 9
Thigh circumference (cm)	56 ± 5	53 ± 7
ST (mm)	14.1 ± 5.8	17.8 ± 8.6

### Arterial occlusive pressure

Data are presented as mean ± SD. AOP was statistically significantly different in the different body positions, *x*^2^(2) = 90.04, *p* < 0.001. Post hoc analysis revealed that AOP in the supine position was significantly lower compared to the seated position (187.00 ± 32.5 vs 204.00 ± 28.5 mmHg, respectively, *p* < 0.001), and the standing position (187.00 ± 32.5 vs 241.50 ± 49.3 mmHg, respectively, *p* < 0.001). Additionally, AOP in the seated position was significantly lower than in the standing position (204.00 ± 28.5 vs 241.50 ± 49.3 mmHg, respectively, *p* < 0.001).

### Test-retest reliability

For the supine position, the ICC for assessing reliability of the device across two repeated measurements was 0.982 (95% CI [0.932–0.995]) with a COV of 2.94% (95% CI [1.90–3.98]). For the seated position, the ICC for assessing reliability of the device across two repeated measurements was 0.975 (95% CI [0.932–0.994]) with a COV of 1.82% (95% CI [0.95–2.69]). For the standing position, the ICC for assessing reliability of the device across two repeated measurements was 0.953 (95% CI [0.822–0.988]) with a COV of 2.97% (95% CI [0.89–5.05]).

## Discussion

This study investigated the influence of body position on AOP measurement. The main findings were that lower-limb arterial AOP is body position dependent. For absolute occlusion of lower-limb arterial blood flow, it appears that higher pressures are required in a seated compared to supine body position, and higher pressures are required in a standing compared to seated and supine body position.

The pressure required to fully restrict arterial blood flow to the lower-limb increased from 187 to 204 to 241 mmHg in the supine, seated and standing positions, respectively. This reflects literature demonstrating increases in peripheral blood flow to the extremities (Goetz, 1950) and changes in hydrostatic pressure with different body positions (Wilkins, Halperin & Litter, 1950; Eiken, 1988). Elevation of the heart above the limbs when comparing a seated to supine position results in an increase in peripheral blood flow and pooling of blood in the lower limbs due to gravitational forces (Olufsen et al., 2005). Increases in lower-limb local hydrostatic pressure (Wilkins, Halperin & Litter, 1950), mechanical deformation of the vascular bed and stimulation of group III afferent fibres (Trinity et al., 2011) triggers peripheral vasodilation, causing a rise in peripheral blood flow. As there is greater elevation of the heart again in a standing position and a larger effect of gravity, these changes in peripheral blood flow and pressure may be amplified further in a standing position (Olufsen et al., 2005). Studies examining factors influencing AOP in the upper-limb support this, with systolic blood pressure identified as one of the major predictive variables of AOP in the upper-limb (Loenneke et al., 2015; Jessee et al., 2016).

### Implications for BFRE

These observations suggest that measurement of AOP and application of the occlusive stimulus in different positions would result in undesirable levels of occlusion, which has important implications for application. For example, if AOP is measured whilst standing but occlusion is applied in a seated or supine position, the individual may be exposed to higher levels of occlusion than necessary. Within BFRE, higher pressures have been shown to cause greater CV responses to exercise (Rossow et al., 2012) and may result in full restriction of arterial inflow to the working muscle. It has been speculated that this may increase the risk of ischemic reperfusion injury, peripheral nerve injury or concerning hemodynamic alterations (Kacin et al., 2015; Loenneke et al., 2011; Jessee et al., 2016), particularly when BFRE is used in patients with blood-related conditions such as hypertension and heart disease (Madarame et al., 2010; Cezar et al., 2016). Additionally, although the focus of this study was not on the physiological responses to BFR, higher pressures are known to increase discomfort responses to BFRE (Jessee et al., 2017; Mattocks et al., 2017) and thus could impact upon the clinical utility of BFRE training and patient adherence to clinical rehabilitation programmes. Therefore, accurate calculation of AOP for pressure prescription is required for selection of the minimum occlusion pressure required to elicit a positive change. It is of note that optimal occlusion pressure is not fully understood, and may vary in different contexts. However, current literature suggests that light-load BFRE (<30% 1 Repetition maximum (RM)) training protocols benefit from higher occlusion pressures (80% vs 40%) (Lixandrão et al., 2015), which would support the importance of accurate AOP measurement for prescription of relative pressures. In contrast when loads are ≥30% 1RM there does not appear to be a need to exercise at higher percentages of AOP (Counts et al., 2015).

On the contrary, measurement of AOP in a supine position and subsequent application of BFRE in a seated or standing position may result in a lower level of occlusion than desired, or a lack of venous occlusion altogether in situations where low pressures are used (Kubota et al., 2011). Furthermore higher pressures during BFRE result in greater accumulation of metabolic by-products (Yasuda et al., 2010) hypothesised to be one of the major driving forces of hypertrophic adaptations to light-load BFR training (Pearson & Hussain, 2015; Hughes et al., 2017). Insufficient levels of restriction due to inaccurate pressure prescription confounded by body position may reduce the metabolic stress stimulus, which may dampen the hypertrophic BFR stimulus and partially explain reports of ineffectiveness of BFRE. Furthermore AOP may be influenced by time of day, with increased pressure observed as the day progresses, likely brought about by oscillatory changes in changes in blood flow and pressure (Ingram et al., 2017). Therefore, measurement position and time of day should be considered by those using BFRE over repeated applications.

### Implications for IPC

When performing IPC before exercise, the lower or upper-limb is occluded at a set arbitrary pressure between 200 and 220 mmHg (Bailey et al., 2012; Barbosa et al., 2014; Patterson et al., 2015). Furthermore, when applying this pressure, participants are either supine (Patterson et al., 2015) or in a seated position (Marocolo et al., 2017), which may influence the amount of occlusion observed. In the current study, the average pressure observed in the supine position was <200 mmHg; however, in the seated position this increased to 204 mmHg. This suggests that the normal arbitrary pressures of 220 mmHg should be sufficient to fully restrict blood flow prior to this intervention. However, in some studies the pressure used has been 200 mmHg (Barbosa et al., 2014) and as low as 180 mmHg (Cunniffe et al., 2016). In the current investigation, 28% and 18% of the participants would not be fully occluded at 200 and 220 mmHg in the supine position, respectively. Furthermore, in the seated position, this number would rise to 60% and 28% for 200 and 220 mmHg, respectively. Therefore, we recommend the use of AOP to standardise pressures during IPC due to the wide variance in participants and also the wide array of cuffs used to occlude individuals.

### Reliability

When measuring AOP automatically, it is important that the device used is reliable and consistent across repeated measures to ensure correct prescription of pressure. In this study, the pneumatic tourniquet system appeared to have high reproducibility (>0.953) with a COV of less than 2.97% across all the body positions examined. These findings are similar to a recent study examining the reliability of Doppler ultrasound for calculating total AOP in the upper limbs (Bezerra de Morais et al., 2016). The authors calculated AOP using Doppler ultrasound in 13 male volunteers across three repeated measures, reporting an ICC of 0.795 and a COV of 5.6%. Although the present study was in the lower-limbs, we observed greater ICC scores and smaller COVs, suggesting measurement of AOP using the pneumatic tourniquet system may be more reliable than Doppler ultrasound. This is may be attributed to the absence of human error; however, this is speculative at present. Nevertheless, other studies have demonstrated similar results. The results of the present study suggest the pneumatic tourniquet system is highly reproducible for measuring lower-limb arterial AOP due to the high ICC values and lower COV scores compared to similar studies in the upper-limb (Bezerra de Morais et al., 2016).

## Conclusion

The findings of the present study have several important clinical implications. Firstly, it appears that AOP is body position dependent. In BFRE and IPC, AOP must therefore be calculated in the position of exercise to ensure accurate occlusion, while minimising the risk of an adverse CV/neurological event or application of an insufficient BFRE stimulus. Secondly, it appears that the pneumatic tourniquet system can be used to reliably calculate lower-limb AOP. Previously, we highlighted that AOP may change across the duration of a BFRE training study due to various tissue adaptations, such as increases in muscle mass and vasculature adaptations, thus it is important to continually monitor AOP to ensure prescription of the correct pressure (Hughes et al., 2017). Doppler ultrasound tools can be expensive, and repeated measurement of AOP using this technique prior to weekly BFRE training sessions would likely be time-consuming and require considerable skill. This may be exacerbated in a clinical rehabilitation setting such as the NHS where rehabilitation exercise classes are already time-constrained. However, the pneumatic tourniquet system provides a simple and quick alternative for calculating AOP, and may be implemented on a session-to-session basis. We propose that an actual measurement of AOP is obtained at rest, prior to BFRE, and a percentage of that measurement is used provide a more reliable stimulus (Laurentino et al., 2012; Hughes et al., 2017; Patterson et al., 2017) as this method is still under-utilised by practitioners (Patterson & Brandner, 2017).

In conclusion, the results of this study indicate that for accurate prescription of occlusion pressure in BFRE and IPC applications, body position must be accounted for an AOP measured in the position that the occlusion stimulus will be subsequently applied. Moreover, the pneumatic tourniquet system appears to have high reproducibility for automatic measurement of AOP in the lower-limbs.

## Supplemental Information

10.7717/peerj.4697/supp-1 Supplemental Information 1 Raw data set for AOP. Raw data for LOP measurement in different body positions.

## Additional Information and Declarations

### Data Availability

The following information was supplied regarding data availability: The raw data are included as a Supplemental File.
